# Supplementary figures and images for: Exogenous Nucleotides Improved the Oxidative Stress and Sirt-1 Protein Level of Brown Adipose Tissue on Senescence-Accelerated Mouse Prone-8 (SAMP8) Mice
Source: Nutrients. 2022 Jul 7;14(14):2796. doi: 10.3390/nu14142796 (PMC9320366; doi:10.3390/nu14142796)

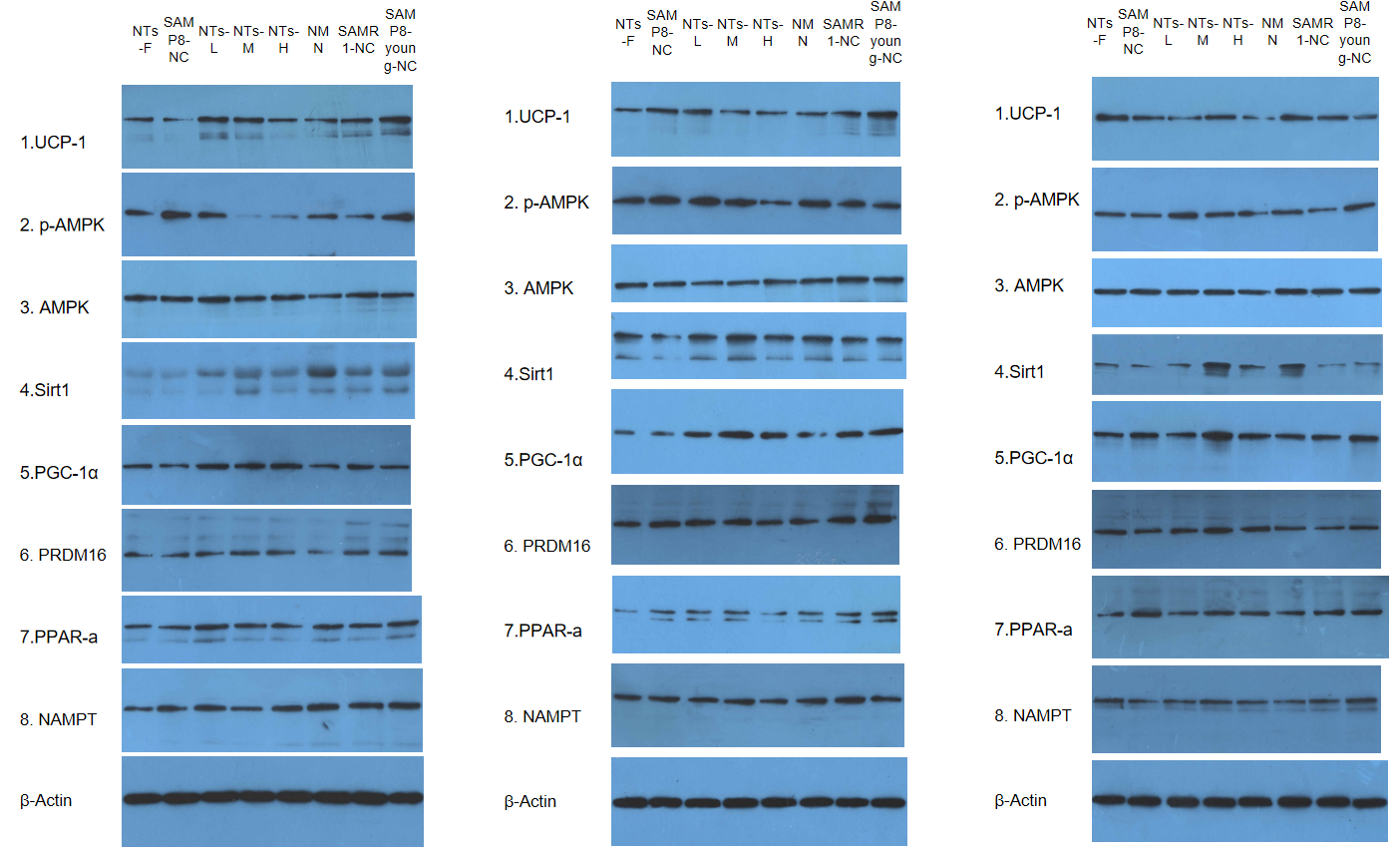

Supplement: Supplementary file 1 [file nutrients-14-02796-s001.zip › nutrients-1682681-supplementary-Figure S1.png]
